# Optimizing Anticorrosion Coating Performance: Synthesis of Polyurethane/Epoxy Hybrids

**DOI:** 10.3390/polym17111516

**Published:** 2025-05-29

**Authors:** Lyazzat Bekbayeva, El-Sayed Negim, Khaldun M. Al Azzam, Rinat Zhanibekov, Gulzhakhan Yeligbayeva, Gulnaz Zhaksylykovna Moldabayeva, Ewies F. Ewies

**Affiliations:** 1 School of Chemical Engineering, Kazakh British Technical University, 106 Walikhanov Street, Almaty 050010, Kazakhstan; a.negim@kbtu.kz (E.-S.N.); rin_zhanibekov@kbtu.kz (R.Z.); 2 National Nanotechnology Open Laboratory, Al-Faraby Kazakh National University, Almaty 050040, Kazakhstan; 3 Department of Chemistry, Faculty of Science, The University of Jordan, Amman 11942, Jordan; k.alazzam@ju.edu.jo; 4 Institute of Organic Synthesis and Carbon Chemistry of the Republic of Kazakhstan, Alikhanov Street., 1, Karaganda 100000, Kazakhstan; g.yeligbayeva@satbayev.university; 5 School of Petroleum Engineering, Satbayev University, 22 Satpayev Street, Almaty 050013, Kazakhstan; g.moldabayeva@satbayev.university; 6 Organometallic and Organometalloid Chemistry Department, Chemical Industries Research Institute, National Research Centre, El-Bhouth Street, Dokki, Giza 12622, Egypt; ef.ewies@nrc.sci.eg

**Keywords:** polyurethane, hybrid, corrosion, epoxy, mechanical, resistance

## Abstract

Corrosion-resistant coatings are essential for prolonging the lifespan of metal structures, yet conventional formulations often lack sufficient mechanical strength and chemical durability. This study focuses on the development of polyurethane/epoxy hybrid coatings (PUAE) with varying epoxy resin content (5%, 10%, and 15% by weight) to enhance performance. The hybrid films demonstrated improved mechanical properties with increasing epoxy content, including a rise in tensile strength from 39.1 MPa (PUA) to 86.3 MPa (PUAE15) and adhesion from 2.5 MPa to 8.3 MPa. Hardness also increased from 69 Shore A to 98 Shore A, while elongation at break decreased from 158% to 95%, indicating a shift toward a stiffer material. The thermal stability, assessed by TGA, showed higher degradation temperatures, with PUAE15 reaching a maximum decomposition temperature of 390 °C, compared to 320 °C for pure polyurethane. Viscosity at 5 rpm increased from 12.300 mPa·s to 18.563 mPa·s, and the contact angle improved from 105° to 149°, highlighting enhanced hydrophobicity. PUAE15 also displayed superior resistance to solvents and acidic environments. These results affirm that epoxy content significantly influences the structural, mechanical, and corrosion-resistant properties of polyurethane-based coatings, making PUAE15 a promising candidate for advanced anticorrosive applications.

## 1. Introduction

Polyurethanes (PUs) are a highly versatile class of polymers widely used in coatings, adhesives, elastomers, and foams due to their excellent toughness, flexibility, abrasion resistance, and strong adhesion to various substrates [1]. Polyurethane is synthesized through a polyaddition reaction between polyols—compounds containing multiple hydroxyl groups—and isocyanates, which feature reactive –N=C=O groups. This reaction results in the formation of urethane linkages, imparting polyurethane with its diverse mechanical and chemical properties. Polyurethane prepolymers can be categorized into aliphatic, aromatic, and cyclic aliphatic types, depending on the nature of the isocyanates used. Additionally, polyols play a crucial role in determining the flexibility of the final polyurethane material, with polyether polyols enhancing elasticity and polyester polyols contributing to rigidity. Using various diiscyanates and polyols, these properties can be modulated over a broad range to suit specific applications [1,2,3]. However, despite their many advantages, polyurethanes have limitations associated with limited thermal stability, lower stiffness, and sensitivity to hydrolysis, particularly in humid or high-temperature environments [4]. Therefore, polyurethanes are generally unsuitable in cases where chemical and thermal stability, as well as high mechanical performance, are critical. Meanwhile, epoxy resins are thermosetting polymers valued for their high strength, chemical resistance, and excellent adhesion, making them indispensable in coatings, adhesives, composites, and electronics. They are typically synthesized through the reaction of epichlorohydrin with bisphenol A (BPA) or other polyphenolic compounds in an alkaline medium. This reaction progresses to form linear oligomers with epoxy functionality, and the molecular weight of the resin can be precisely controlled by adjusting the BPA-to-epichlorohydrin ratio [5,6].

To address the limitations of PUs, they are frequently combined with epoxy resins in epoxy–polyurethane combination coatings [7,8,9]. Epoxy resins are thermosetting polymers known for their high mechanical properties, excellent chemical and thermal resistance, and superior adhesion properties [10,11]. However, they are inherently brittle and lack flexibility and impact resistance compared to PUA systems [12,13]. In addition, pure epoxy resins have poor UV resistance, resulting in yellowing and surface degradation over time [14]. The layered approach in epoxy–polyurethane composites effectively mitigates the individual drawbacks of each material, resulting in a system with enhanced durability and performance. However, this type of coating is associated with high cost, complex application procedures, and potential adhesion issues between layers [15,16]. 

Another advanced approach is EP/PUA hybrid systems, which have garnered significant interest due to their potential to combine the advantageous properties of both materials in one matrix [17,18]. The hybridization of these two systems offers a strategic approach to overcoming the limitations of each, yielding materials with tailored mechanical, thermal, and processing properties. These hybrids may be synthesized via various routes, including graft-polymerization, chain extension, crosslinking, or simultaneous curing, depending on the reactive functional groups present [19,20]. The resulting materials often exhibit enhanced tensile strength, shape memory behavior, or improved chemical resistance compared to single-component systems [21]. Depending on the synthesis conditions, EP/PU hybrids may contain different functionalities. Chung et al. [19] conducted the synthesis of an epoxy–polyurethane hybrid by graft-polymerizing a bisphenol A/epichlorohydrin-based epoxy onto a polyurethane backbone, which resulted in a covalently bonded network with enhanced tensile strength and shape-recovery properties, though low-temperature flexibility remained unchanged. Chen et al. [20] obtained an EP/PU interpenetrating network using frontal polymerization, a method that allowed rapid and energy-efficient curing while maintaining structural and thermal properties comparable to conventional approaches. Another EP/PU hybrid composite was prepared by Chen et al. [22] through incorporation of silane-modified nano-SiO_2_ and Al_2_O_3_ particles into the polymer matrix, which significantly improved mechanical strength and electrical insulation performance. The incorporation of 10 wt% optimally mixed inorganic nanofillers (SiO_2_: Al_2_O_3_ = 4.5:5.5) into the EP/PU matrix produced a composite with notably improved properties, achieving ~28.5 MPa shear strength and a 15 kV/mm dielectric breakdown field. Peng et al. [23] synthesized a recyclable EP/PU hybrid using Diels–Alder chemistry to form reversible covalent bonds between functionalized epoxy and polyurethane chains, yielding a material with excellent mechanical strength and the ability to be thermally reprocessed without degradation. Ghozali et al. [24] suggested a simultaneous incorporation of polyol, isocyanate, and epoxy resin to form a more compatible hybrid matrix. The synthesized hybrids, prepared without prepolymer intermediates, demonstrated improved mechanical properties and varying thermal stability depending on the type of polyol used. Similarly, in [25], a hybrid nanocomposite was synthesized by blending epoxy resin with polyurethane prepolymer and reinforcing it with functionalized multi-walled carbon nanotubes and SiO_2_ nanoparticles, resulting in significantly improved hardness, elastic modulus, and wear resistance. Kausar [26] proposed a strategy based on the physical interlocking of polymer chains, which can improve compatibility but may compromise overall mechanical properties unless reinforced properly. There are several studies related to the development of so-called non-isocyanate polyurethane epoxy hybrid materials, in which isocyanates are not used directly for polymer synthesis. These systems are typically produced via the reaction of cyclic carbonates—often obtained from CO_2_—with amines to form polyhydroxyurethanes, which are then crosslinked with epoxy resins. This approach allows for the creation of hybrid networks with reduced toxicity, improved environmental sustainability, and tunable properties such as mechanical strength, chemical resistance, and thermal stability [27,28]. Wang et al. [29] reported an epoxy-free synthesis of aromatic five-membered cyclic carbonates and their conversion into strong epoxy hybrid non-isocyanate polyurethanes. These materials exhibited excellent mechanical properties (tensile strength up to 45.1 MPa), high crosslinking density, and improved thermal and swelling resistance due to the incorporation of aromatic segments [30].

This paper presents research findings on enhancing the mechanical properties of polyurethane polymers incorporating epoxy resin. It further explores the optimization of these coatings to improve chemical and solvent resistance, specifically for metal substrates.

## 2. Materials and Methods

### 2.1. Materials

Polypropylene glycol (PPG) with varying molecular weights and hydroxyl (OH) numbers were utilized as follows: PPG (Mw = 2000 g/mol, OH number = 56 mg KOH/g, Korea PTG, Seoul, Republic of Korea), PPG (Mw = 1200 g/mol, OH number = 98 mg KOH/g, Korea PTG, Republic of Korea), and PPG (Mw = 2700 g/mol, OH number = 37 mg KOH/g, Korea PTG, Seoul, Republic of Korea). Epoxy YD128 was used (unmodified liquid epoxy resin based on bisphenol-A with medium viscosity, Mw = 700 g/mol, water content 0.05% max, Kukdo Chemical Co., LTD, Seoul, Republic of Korea). Prior to use, all PPG and YD128 samples were dried and degassed at 80 °C under a vacuum of 1–2 mmHg for 2 h. Other materials employed in the study include Dibutyltin dilaurate (DBTDL, Fluka, Chemie, UK) and 1,1′-Methylenebis(4-isocyanatobenzene) (MDI, Bayer AG). The following chemical reagents were sourced from Aldrich: NaCl (10% solution), NaOH (1 M), HCl (1 M), H_2_SO_4_ (1 M), and HNO_3_ (1 M). Solvents such as acetone, xylene, toluene, benzene, butanol, isopropyl alcohol, chloroform, and cyclohexane were obtained from Fluka (Chemie, UK). For the formulations, titanium dioxide pigment (Tiona-595, crystal, Al Jubail, Saudi Arabia) was incorporated along with rheological and anti-setting additives—SR882 and Bentone 27 (Elementis, Kuala Lumpur, Malaysia). The dispersing agent used was Troysperse CD1 (Troy, ON, Canada).

### 2.2. Synthesis of the Prepolymer Polyurethane (PUA)

The polyols (PPG-2000, PPG-1200, and PPG-2700) were introduced into a 500 mL round-bottom, three-necked separable flask, equipped with a mechanical stirrer, thermometer, and a condenser fitted with a drying tube. The reaction was conducted in a nitrogen atmosphere using a constant-temperature oil bath. MDI was then added to the flask, and the mixture was heated at 95 °C for 1 h. Subsequently, YD128 (E) was introduced, and the reaction was continued at the same temperature until the theoretical NCO value was achieved, as determined by the di-n-butylamine titration method (ASTM D 2572-19) [31]. The resulting samples formed a viscous prepolymer.

**Scheme 1:**
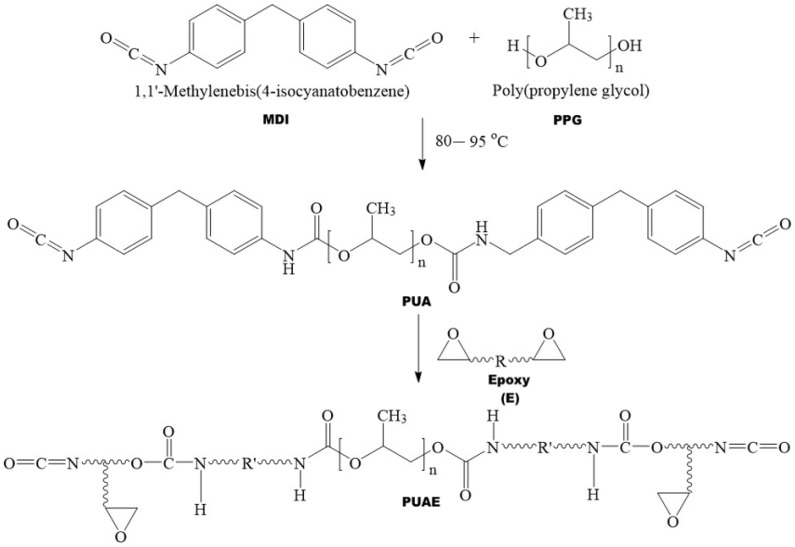
The synthesis and structural development of polyurethane (PUA) and its hybrid formulation with epoxy (PUAE).

The reaction scheme for the prepolymer synthesis is depicted in Scheme 1, and samples with varying E contents while maintaining constant NCO/OH ratios are detailed in Table 1.

### 2.3. Preparation of Polyurethane Coatings (PUAC and PUAEC)

**Figure 1:**
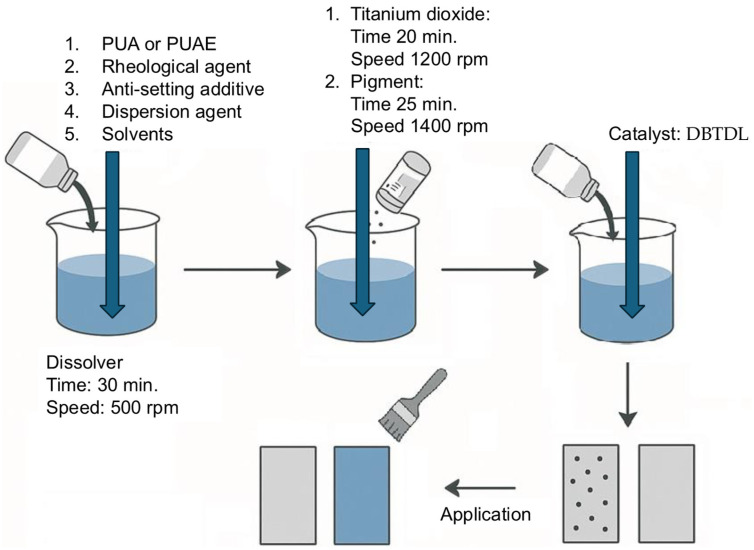
Preparation of the polyurethane coatings PUAC and PUAEC. Please refer to Section 2.3 for the conditions used.

Table 2 presents the weight percentages of the components incorporated into polyurethane coatings for anticorrosion purposes. Initially, the rheological agent, dispersion agent, and anti-settling additives were mixed with the prepolymer (PUA or PUAE) using a 2 L dissolver at 500 rpm for 30 min. Subsequently, titanium dioxide was introduced into the mixture and stirred for 20 min at 1200 rpm. Following this, the pigments were incrementally added and homogenized with solvents (xylene and butanol) for 25 min at 1400 rpm, adjusting the viscosity to 3000 ± 500 cps (Figure 1). Lastly, the catalyst, DBTDL, was applied during the coating process onto metal surfaces.

### 2.4. Film Coating Preparation

The coating samples (CPUA or CPUAE) were applied to mild steel panels (70 mm × 200 mm) in accordance with ASTM D4147-18 [32] standards and were left to dry at room temperature for 7 days. Mild steel is a type of low-carbon steel, typically containing carbon (0.05–0.25%), Mn (0.25–0.90%), Si (up to 0.40%), S (<0.05%), P (<0.05%), and Fe (major component). The dried films were subsequently stored in a desiccator at ambient conditions for further characterization and measurements. The thickness of the coatings was determined to be within 70–75 µm in the dry state and 80–90 µm in the wet state, using the Sheen-Ecotest Plus B FN2 device, model 121-17-00.

### 2.5. Characterization of the Prepolymer Polyurethane (PUA and PUAE)

The synthesized PUA and PUAE were analyzed using ALPHA Fourier Transform Infrared (FTIR) spectroscopy (Bruker, Billerica, UK) to determine the functional groups of PUA and PUAE. Thermogravimetric analysis (TGA) was conducted to evaluate the thermal properties of the dried terpolymers. Using a Perkin Elmer TGA/SDTA851e (Perkin Elmer, WA, USA), samples were analyzed from ambient temperature to 800 °C at a heating rate of 10 °C per minute in an air atmosphere. Each measurement utilized an 8 mg sample, with weight loss recorded as a function of temperature. Their viscosities (mPa·s) were measured at room temperature using a Brookfield viscometer, adhering to ISO 12058-1 [33] standards, at rotational speeds of 5 and 50 rpm.

### 2.6. Mechanical Tests for PUA, PUAE, PUAC, and PUAEC

The tensile properties of the PUA, PUAE, CPUA, and CPUAE films were evaluated using an MTS 10/M tensile testing machine at a crosshead speed of 50 mm/min, employing a 1-kN load cell with an average of at least four measurements recorded. Shore A hardness was determined with an indentation hardness tester following ASTM D2240-15 [34] standards. Contact angles between water droplets and the sample surface were measured using a CAHN DCA-322 analyzer at 25 °C, with a droplet velocity of 100 µm/s. The resistance to cracking or detachment of coatings from metal substrates under bending stress was tested using a cylindrical Mandrel Tester (ASTM D522) [35]. Impact resistance was assessed via a tubular impact tester (ASTM D2794) [36], while adhesion of the coatings was evaluated with an economic crosshatch tester (ASTM D3359) [37]. For adhesion strength, pull-out tests of PUA, PUAE, CPUA, and CPUAE were conducted according to standardized methods (EN 1542) [38].

### 2.7. Corrosion Resistance Tests for PUAC and PUAEC

Corrosion resistance tests were performed on coated panels exposed to salt (10% NaCl), base (1.0 M NaOH), and acid (1.0 M HCl). Additionally, solvent resistance (ASTM D5402-19) [39] and water resistance (ASTM D1647-89) [40] tests were carried out. The corrosion test was undertaken at a specific time for one week. Dry times were documented at an ambient temperature of 25 °C.

## 3. Results and Discussion

### 3.1. FTIR Analysis

**Figure 2:**
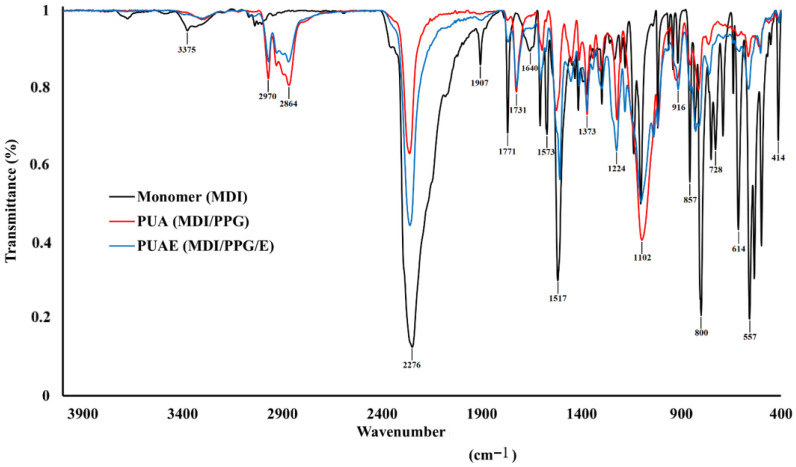
FTIR spectrum of monomer MDI, PUA, and PUAE.

The addition polymerization involving isocyanate (NCO) and polyol (OH) was evaluated through FTIR spectroscopy, as shown in Figure 2. In the FTIR spectrum of PUA (Figure 1), peaks observed in the 3000–3150 cm^−1^ range are associated with N–H stretching, while peaks at 2969–2922 cm^−1^ signify C–H stretching. Vibrations at 1517 cm^−1^ were attributed to N–H bending, while the absorption band at 1102 cm^−1^ corresponded to C–O–C bonds. Additional absorption peaks at 1640 cm^−1^ and 1454 cm^−1^ were linked to the benzene rings in MDI. A new peak at 1731 cm^−1^ was identified, corresponding to carbonyl group stretching. This peak emerged from the reaction between NCO groups from MDI and OH groups present in polyols (PPG 120, 2000, 2700). The NCO peak at 2276 cm^−1^ showed a significant intensity reduction—over 60%—indicating its reaction with polyols (OH) during the polyaddition process [41]. Meanwhile, the FTIR spectrum of PUAE highlighted a new peak at 940 cm^−1^, assigned to epoxy group stretching in E as shown in Figure 2. The NCO peak initially observed at 2243 cm^−1^ decreased from 95% for monomer MDI to 55% for PUAE (Figure 1) and decreased to 35% at 2261 cm^−1^ for PUA, signifying the consumption of OH groups from PPG and epoxy (E) in approximately 50% of the NCO groups during the addition polymerization as shown in Scheme 1. The literature widely acknowledges that the infrared absorbance of hydrogen-bonded urethane or urea carbonyl occurs at a lower wavenumber compared to free carbonyl. In the case of PUA and PUAE, the two overlapping bands at 1767 and 1724 cm^−1^ correspond to the free and hydrogen-bonded urethane C=O groups, respectively [42].

### 3.2. TGA Analysis

**Scheme 2:**
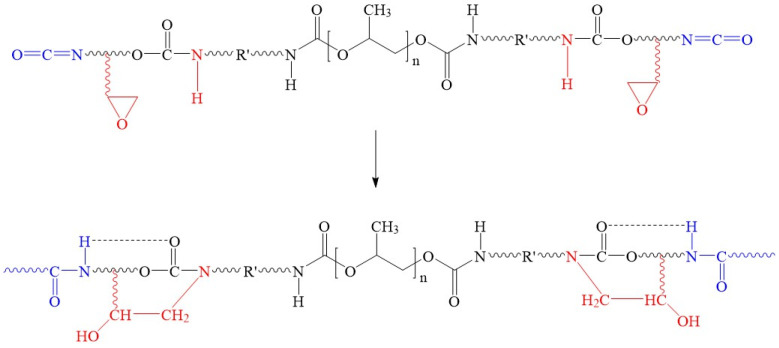
The interaction and bonding mechanisms between polyurethane (PUA) and epoxy (E).

TGA was performed to determine the thermal stability of PUA and PUAE under a nitrogen atmosphere using a Perkin Elmer TGA/SDTA851e thermogravimeter. Table 3 shows three degradation stages for PUA and PUA hybrid with 5% and 10% E, while the degradation of PUA hybrid with 15% was one step. The initial weight loss was 2.7% for PUA at a temperature of 150 °C, increased to 3.1% for PUAE5 at 160 °C and 3.7% for PUAE10 at 174 °C. The slight initial loss of weight due to the vaporization of residual and linked solvent is shown in Supplementary Figure S1. As shown in Table 3, the thermal stability of films in the second stage is increased with polyurethane hybrids (PUAE). For example, the weight loss in the second stage was 92% for PUA at 545 °C, while the weight loss for PUAE5 was 91.5% at 535 °C and 80.2% for PUAE10 at 407 °C. The increase in the thermal stability of the polyurethane hybrid is attributed to crosslinking between different functionalities between polyurethane and epoxy [43], as shown in Scheme 2. The weight loss in the third stage of degradation was 3.6% for PUA at 819 °C, 5.1% for PUAE5 at 845 °C, and 15.1% for PUAE10 at 897 °C. However, the polyurethane hybrid with 10% epoxy content showed one stage of degradation at temperatures of 39–786 °C with weight loss of 99.9 wt. As the epoxy content increased in the polyurethane hybrid, the initial decomposition and maximum polymer degradation temperatures (PDT_max_) increased, as shown in Table 3. PUAE15 with 15% epoxy content, showed the highest PDT_max_ at a temperature of 390 °C as compared to PUA (320 °C). The enhanced thermal stability and mechanical strength of polyurethane can be attributed to the influence of epoxy and its content, which modifies the polymer structure and increases crosslink density [44,45,46], as illustrated in Scheme 2. Research indicates that epoxy–polyurethane composites exhibit superior thermal resistance, and epoxy grafting onto polyurethane can further alter its degradation behavior, potentially minimizing weight loss at elevated temperatures [47,48].

### 3.3. Viscosity of PUA and PUAE

**Figure 3:**
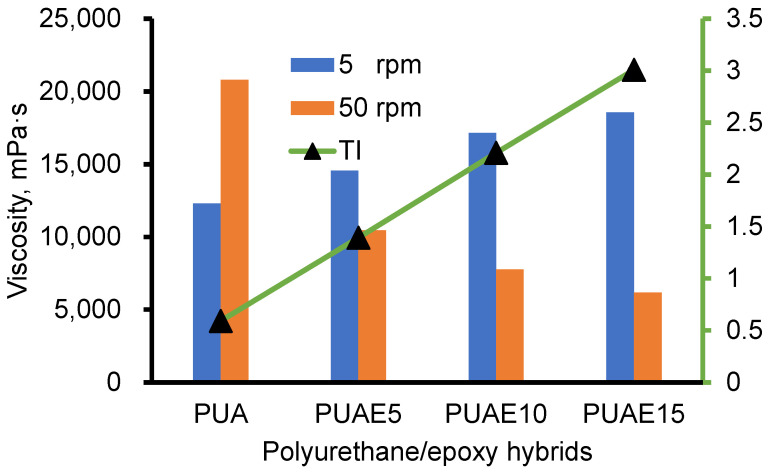
Viscosity and thixotropic index of PUA and PUAE: effect of epoxy content variations.

The viscosity of a polymer is a critical factor in its application within the coating industry. High viscosity poses application challenges, while low viscosity can lead to sagging issues. The effect of epoxy (E) content on the thixotropic index (TI) and viscosity of PUA at 5 and 50 rpm is illustrated in Figure 3. At 5 rpm, the viscosity of PUA increased from 12.300 mPa·s to 14.560 mPa·s for PUAE5 (5% epoxy), to 17.150 mPa·s for PUAE10 (10% epoxy), and to 18.563 mPa·s for PUAE15 (15% epoxy). Conversely, at 50 rpm, the viscosity of PUA decreased from 20.800 mPa·s to 10.456 mPa·s (PUAE5), 7763 mPa·s (PUAE10), and 6171 mPa·s (PUAE15). These changes arise from the effect of epoxy on the soft segment of polyurethane, which is closely associated with the polyol backbone. This interaction leads to the formation of additional hydroxyl groups, strengthening hydrogen bonding and intermolecular forces, ultimately resulting in increased viscosity [49,50,51,52]. The thixotropic index (TI) was also affected by epoxy content, as shown in Figure 3. The addition of epoxy significantly enhanced the TI, which increased from 0.59 for PUA to 1.39 for PUAE5 and reached 3.1 for PUAE15.

### 3.4. Mechanical Properties

Mechanical properties, including tensile strength, elongation at break, hardness, and adhesion, are critical parameters for the practical application of polymers. The effect of epoxy content on the mechanical properties of PUA is summarized in Table 4. As shown in Table 4, the addition of epoxy to PUA, forming PUA/E hybrids, led to an increase in tensile strength, hardness (shore A), and adhesion, accompanied by a decrease in elongation at break. This improvement in tensile strength, hardness, and adhesion is attributed to the crosslinking of epoxy with PUA via the ring-opening reaction of epoxy groups, which forms a crosslinked network, as illustrated in Scheme 2. With an increase in epoxy content from 5% to 15% in the PUA/E hybrids, the tensile strength increased significantly from 53.5 MPa for PUAE5 (5% epoxy content) to 86.3 MPa for PUAE15 (15% epoxy content). Conversely, the elongation at break decreased from 130% for PUAE5 to 95% for PUAE15. Among the hybrids, PUAE15 exhibited the highest hardness (shore A) at 98, while PUAE5 had the lowest hardness at 81. Table 4 illustrates the adhesion characteristics of PUA and the PUA/E hybrids, underlining the influence of epoxy content on the PUA resin’s bonding efficiency with metal substrates. The adhesion of PUA was recorded at 2.5 MPa, which showed significant enhancement upon integrating epoxy within the PUA structure. These hybrids demonstrate distinct characteristics, such as improved adhesion, determined by the component ratio and epoxy resin content [36,39,53]. Increasing the epoxy content in the hybrids from 5% to 15% resulted in adhesion improvement, from 4.5 MPa for PUAE5 to 8.3 MPa for PUAE15. This enhancement is attributed to hydroxyl groups generated during the reaction between PUA and epoxy rings, reinforcing the hybrid’s bonding with metal substrates.

### 3.5. Coating Properties

#### Drying Time and Mechanical Properties

Hybrid coatings with the highest epoxy content demonstrated the shortest pot life and drying time compared to polyurethane coatings, as detailed in Table 5. An increase in epoxy content within the hybrid coating resulted in reduced drying times, encompassing both set-to-touch and dry-hard stages. The reduction in drying time is due to the hydroxyl groups originating from the epoxy and the polyol within the polyurethane’s soft segment [54]. The hybrid coating (CPUAE15) containing 15% epoxy exhibited the shortest drying time, outperforming coatings with a lower epoxy content, such as those with CPUAE5 (5% epoxy content).

The mechanical properties of polyurethane (CPUA) and hybrid coatings with varying epoxy resin content are presented in Table 5. Hybrid coatings (CPUAE) demonstrated higher tensile strength compared to pure polyurethane coatings. This improvement is attributed to crosslinking and hydrogen bonding between urethane, hydroxyl groups, and ester groups, which enhance the mechanical properties [55]. Specifically, the tensile strength of hybrid coatings increased by 27.4% with the addition of 5% epoxy content and by 51.6% with 15% epoxy content. However, as the epoxy content increased, elongation at break decreased. For the polyurethane coating (CPUA), the elongation at break was 243%, which reduced to 221%, 205%, and 197% for hybrid coatings containing 5%, 10%, and 15% epoxy content, respectively.

Table 5 demonstrates the effect of epoxy addition to polyurethane, revealing an increase in adhesion with higher epoxy content. This improvement is attributed to the presence of functional groups—such as hydroxyl, carbonyl, N–H, and epoxy—in the hybrid coating. As the epoxy content increased from 5% to 15%, these functional groups became more prevalent, enhancing the coating’s adhesion to the metal substrate. Specifically, the adhesion strength increases from 7.2 MPa for CPUA to 11.1 MPa for the hybrid coating (CUPAE15) with 15% epoxy content. Additionally, a crosshatch adhesion test was conducted, where adhesive tape was applied to a grid with five cuts in each direction and removed at an angle close to 180°. The results revealed that all samples passed the adhesion test (crosshatch), demonstrating strong bonding to the metal substrate. Additionally, the samples exhibited remarkable flexibility and impact resistance, except for CPUA, which failed in impact resistance. Overall, the increase in hard segments from epoxy resin and NCO/OH contributed to enhanced adhesion and hardness [56]. The hardness of the polyurethane coating improved as the epoxy content increased from 5% to 15%. CUPAE15 exhibited the highest hardness compared to CUPA5 and CUPA. As the functional group content in the hybrid coating increased, its hydrophobicity also improved.

The contact angle serves as an indicator of the coating’s hydrophobic properties. All samples demonstrated hydrophobic characteristics, with contact angles exceeding 100°. With higher epoxy content, the hydrophobic groups in the hybrid coating became more prominent, resulting in an increase in the contact angle from 127° for CUPAE5 to 149° for CPUAE15.

### 3.6. Chemical and Corrosion Resistance

**Figure 4:**
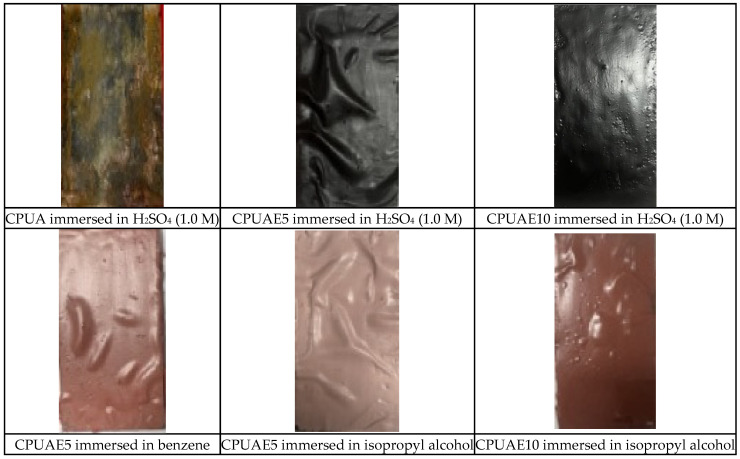
Hybrid coatings immersed in H_2_SO_4_ (1.0 M), benzene, and isopropyl alcohol.

The effect of epoxy content on the chemical and corrosion resistance of CPUA and CPUAE is detailed in Table 5, and all samples remained unaffected by water. However, the CPUA samples were affected by exposure to 1.0 M solutions of HCl, H_2_SO_4_, and HNO_3_. The addition of epoxy to PUA enhanced its corrosion resistance. Specifically, CUPAE10 and CUPAE15 exhibited stability in acid, base, and salt solutions, while CUPAE5 experienced swelling and blistering when exposed to H_2_SO_4_ and HNO_3_, as shown in Figure 4. The CPUA samples failed in all solvent tests except acetone, due to the high proportion of softer segments derived from polyol [20]. Nevertheless, the hybrid coatings demonstrated satisfactory performance during solvent testing and were unaffected across all solvent tests when containing 15% epoxy content. This enhancement is due to the influence of epoxy on the soft segments of polyurethane, leading to the development of harder segments through crosslinked network formation. As a result, permeability to moisture, chemicals, and corrosive agents is significantly reduced [51,52,53,54,55,56,57]. Similar findings have been reported in previous studies [57], indicating that epoxy-modified polyurethane coatings exhibit enhanced resistance to acids, alkalis, and solvents compared to their unmodified counterparts. This effect is particularly beneficial in coating and protective applications. On the other hand, the hybrid coatings with 5% epoxy content swelled in benzene and isopropyl alcohol, whereas those with 10% epoxy content only swelled in isopropyl alcohol, as shown in Figure 4.

## 4. Conclusions

The polyurethane (PUA) and polyurethane/epoxy hybrid (PUAE) coatings were successfully prepared using MDI (isocyanate, NCO) and polyols (OH), including polypropylene glycol (PPG) with varying molecular weights and epoxy content (5%, 10%, and 15% based on polyol) at a 2.5 NCO/OH ratio. FTIR analysis confirmed the reactivity of the NCO peak and provided evidence for urethane formation. The mechanical properties, along with the chemical and corrosion resistance of both the PUA and PUAE coatings, were comprehensively studied. Results revealed that the intermolecular hydrogen bonding between the hydroxyl group of epoxies and the isocyanate group in PUA played a pivotal role in enhancing crosslinking network formation in PUAE. This contributed to improved mechanical properties and adhesion of the hybrid films to the metal substrate. The hybrid coating films showed better mechanical properties and adhesion to the metal substrates than PUA films. As the epoxy content in the hybrid coating formulation increased, notable improvements were observed in mechanical properties as well as chemical and corrosion resistance. Samples with 15% epoxy content exhibited superior thermal stability, along with enhanced mechanical, physical, chemical, and anticorrosive properties in the hybrid coating compared to PUA.

## Figures and Tables

**Table 1:** Feed compositions of the prepolymer polyurethane (PUA) with different contents of polyols.

Samples	PUA		PUAE5		PUAE10		PUAE15	
	Wt (g)	Wt (%)	Wt (g)	Wt (%)	Wt (g)	Wt (%)	Wt (g)	Wt (%)
Polyols, OH								
PP-1200	30.64	20.32	30.64	22,21	30.64	24.10	30.64	25.98
PP-2000	66.39	44.03	46.73	33.87	30.05	23.63	15.85	13.44
PP-2700	13.78	9.15	13.78	9.99	13.78	10.85	13.78	11.69
E	0.0	0.0	6882	5.0	12.72	10.00	17.69	15.00
Mole of OH (gm/mole)	0.06384		0.06384		0.06384		0.06384	
Isocyanate, NCO								
MDI	39.93	26.50	39.93	28.94	39.93	31.41	39.93	33.87
Total	150.74	100	137.98	100	127.15	100	117.91	100
Mole of NCO (gm/mole)	0.1596		0.1596		0.1596		0.1596	
NCO/OH	2.5		2.5		2.5		2.5	

**Table 2:** Anticorrosion polyurethane coating (PUAC) formulations.

Raw Materials	Weight Percent
Prepolymer PUA or PUAE	35
Rheological agent	0.5
Titanium dioxide	30
Anti-settling additive	1.5
Dispersion agent	0.03
Pigment	10.22
Xylene	15
Butanol	6
DBTDL	1.75

**Table 3:** Thermal properties of PUA and PUAE at different contents of epoxy.

Samples	Stages	Decomposition Temperature, °C		Weight Percentage, %	PDT_max_ (°C)
		T1	T2	Δ W	
PUA	1	29	150	2.7	320
	2	150	545	92	
	3	545	819	3.6	
PUAE5	1	29	160	3.1	320
	2	160	535	91.5	
	3	535	845	5.1	
PUAE10	1	30	174	3.7	310
	2	174	407	80.2	
	3	407	897	15.1	
PUAE15	1	39	786	99.9	390

**Table 4:** Mechanical properties of the PUA and PUAE cast films.

	PUA	PUAE5	PUAE10	PUAE15
Tensile strength (MPa)	39.1 ± 1.18	53.5 ± 1.28 ***	64.9 ± 1.22 ****	86.3 ± 1.65 ****
Elongation (%)	158 ± 3.61	130 ± 4.58 ***	112 ± 4.00 ****	95 ± 4.58 ****
Hardness (shore A)	68.3 ± 1.15	81.3 ± 0.58 ****	87.3 ± 1.53 ****	98.0 ± 1.73 ****
Adhesion (MPa)	2.5 ± 0.3	3.8 ± 0.4 **	5.9 ± 0.3 ***	8.3 ± 0.4 ****

Note: A statistically significant difference is given as ****—ρ < 0.0001; ***—ρ < 0.001; **—ρ < 0.01; and no statistically significant difference—ρ > 0.05.

**Table 5:** Mechanical, chemical, and corrosion properties of polyurethane and polyurethane hybrid coating films.

	CPUA	CPUAE5	CPUAE10	CPUAE15
Pot Life and Dry Time				
Pot life (25 °C, min)	105	80	68	51
Dry time (set to touch), h	13	9	8.5	7
Dry time (dry hard), h	22	18	17	15
Mechanical Properties				
Tensile strength (N/m^2^)	62	79	86	94
Elongation (%)	243	221	205	197
Adhesion (MPa)	7.2	8.9	9.9	11.1
Crosshatch (%)	100	100	100	100
Impact resistance (1 m/1 kg)	F	P	P	P
Hardness (Shore A)	77	85	91	96
Conical-Mandrel (¼″)	P	P	P	P
Contact angle	105	127	135	149
Corrosion resistance				
NaCl (10%)	O	O	O	O
NaOH (1.0 M)	O	O	O	O
HCl (1.0 M)	X	O	O	O
H_2_SO_4_ (1.0 M)	X	Δ	O	O
HNO_3_ (1.0 M)	X	Δ	O	O
Water	O	O	O	O
Chemical resistance				
Acetone	Δ	O	O	O
Xylene	X	O	O	O
Toluene	X	O	O	O
Benzene	X	Δ	O	O
Isopropyl alcohol	X	Δ	Δ	O
Chloroform	X	O	O	O
Cyclohexane	O	O	O	O

Note on abbreviations: O = Suitable, Δ = Swelling and blistering, X = Not Suitable, P = Samples passed the test, F = Samples failed the test.

## Data Availability

The original contributions presented in the study are included in the article/Supplementary Materials, further inquiries can be directed to the corresponding author.

## Supplementary Materials

The following supporting information can be downloaded at: https://www.mdpi.com/article/10.3390/polym17111516/s1. Figure S1: TGA Results.
