# Supplementary figures and images for: Optimizing Anticorrosion Coating Performance: Synthesis of Polyurethane/Epoxy Hybrids
Source: Polymers (Basel). 2025 May 29;17(11):1516. doi: 10.3390/polym17111516 (PMC12157741; doi:10.3390/polym17111516)

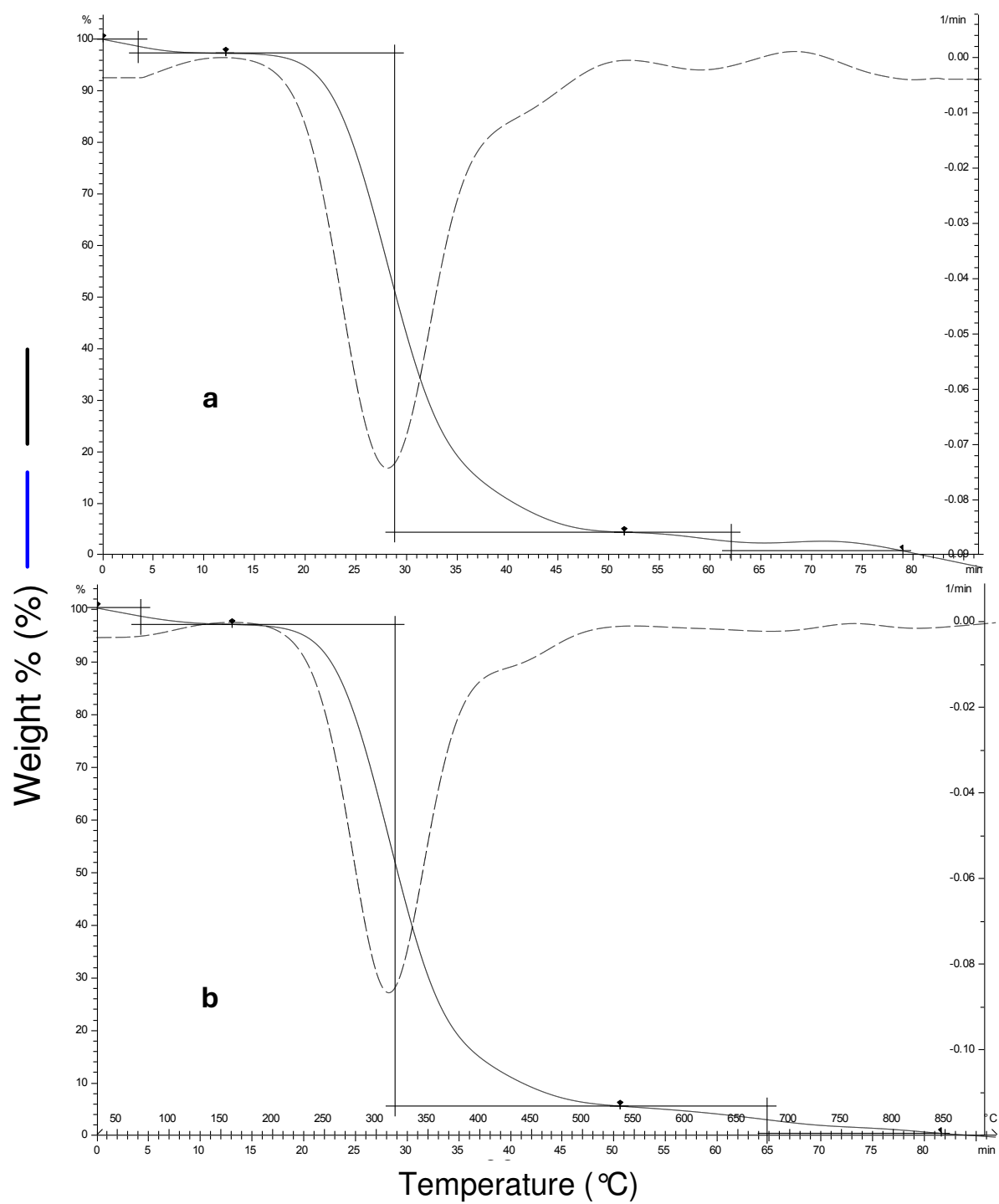

TGA spectra of (a) PUA and (b) PUAE5

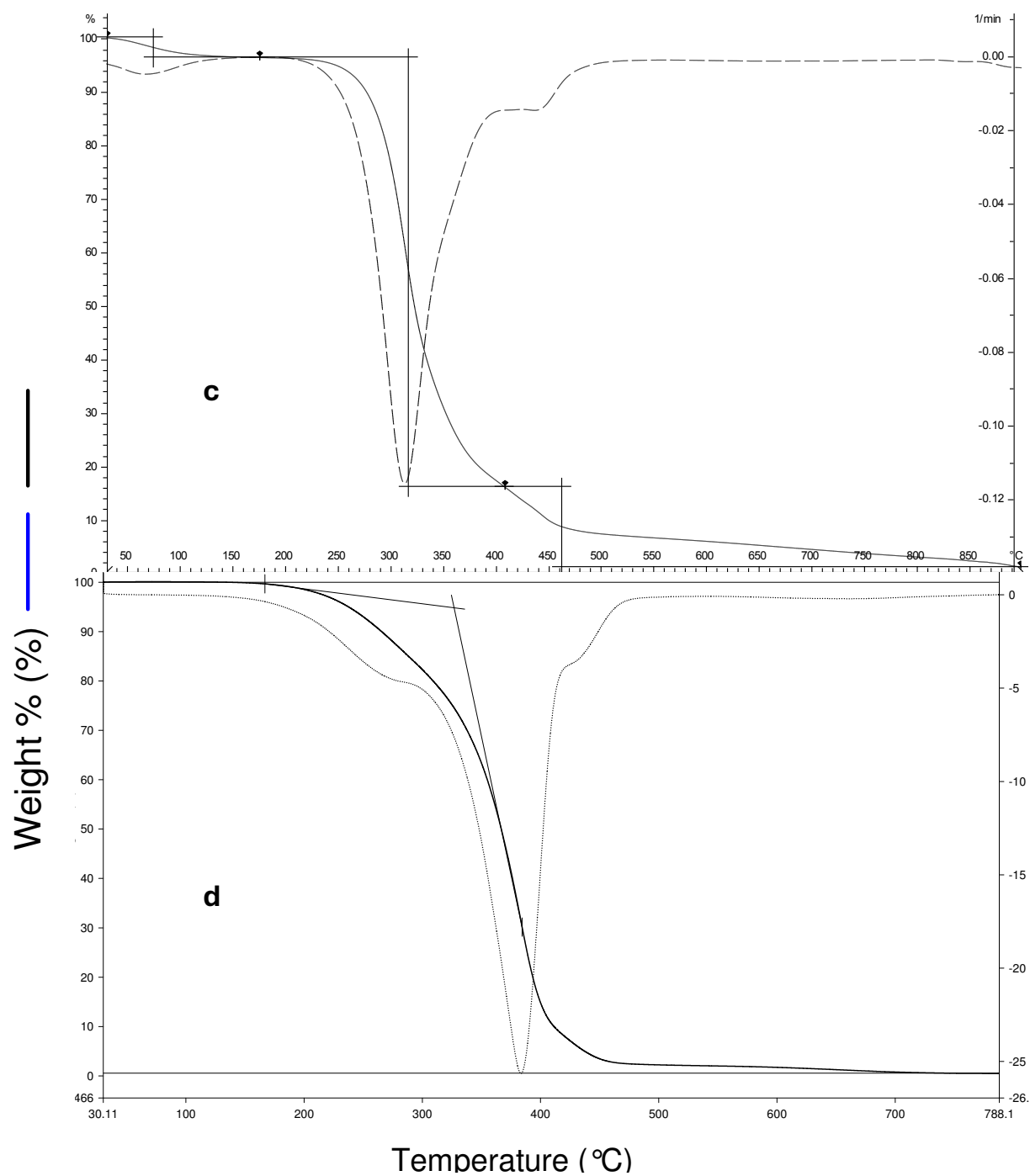

TGA spectra of (c) PUAE10 and (d) PUAE15

Supplement: Supplementary file 1 [file polymers-17-01516-s001.zip › polymers-3631503-supplementary.pdf]
